# Supplementary material for: The causal relationship between the human gut microbiota and pyogenic arthritis: a Mendelian randomization study
Source: Front Cell Infect Microbiol. 2024 Nov 26;14:1452480. doi: 10.3389/fcimb.2024.1452480 (PMC11629706; doi:10.3389/fcimb.2024.1452480)
Supplement: Supplementary file 1 [file DataSheet1.zip › mendelian test/ebi-a-GCST90027623.csv_forest.pdf]

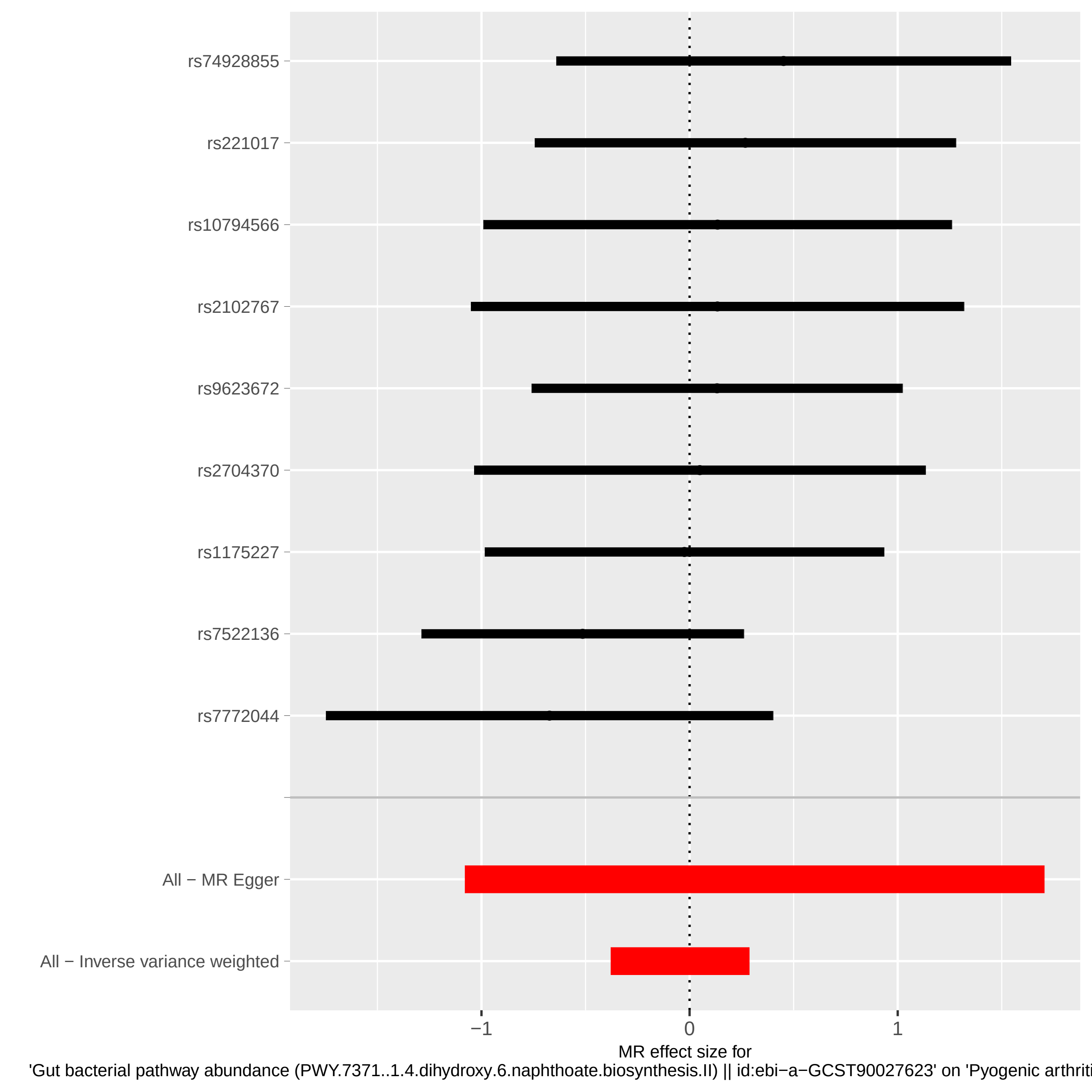
rs74928855
rs221017
rs10794566
rs2102767
rs9623672
rs2704370
rs1175227
rs7522136
rs7772044
All − MR Egger
All − Inverse variance weighted
−1
0
1
MR effect size for
'Gut bacterial pathway abundance (PWY.7371..1.4.dihydroxy.6.naphthoate.biosynthesis.II) || id:ebi−a−GCST90027623' on 'Pyogenic arthriti
